# ‘Alexandrian’ glass confirmed by hafnium isotopes

**DOI:** 10.1038/s41598-020-68089-w

**Published:** 2020-07-09

**Authors:** Gry H. Barfod, Ian C. Freestone, Charles E. Lesher, Achim Lichtenberger, Rubina Raja

**Affiliations:** 1 Aarhus Geochemistry and Isotope Research (AGiR) Platform, Department of Geoscience, Aarhus University, 8000 Aarhus C, Denmark; 2 The Danish National Research Foundation’s Centre of Excellence for Urban Network Evolutions (UrbNet), Aarhus University, Højbjerg, Denmark; 3 UCL Institute of Archaeology, London, WC1H 0PY UK; 4 Institut für Klassische Archäologie und Christliche Archäologie/Archäologisches Museum, Westfälische Wilhelms-Universität Münster, Domplatz 20-22, D-48143, Münster, Germany; 5 School of Culture and Society, Aarhus University, 8000 Aarhus C, Denmark

**Keywords:** Geochemistry, Sedimentology, Tectonics

## Abstract

Archaeological glass contains information about the movement of goods and ancient economies, yet our understanding of critical aspects of the ancient glass industry is fragmentary. During Roman times, distinct glass types produced in coastal regions of Egypt and the Levant used evaporitic soda (natron) mixed with Nile-derived sands. In the Levant, furnaces for producing colourless Roman glass by addition of manganese have been uncovered, whereas the source of the desirable antimony-decolourised Roman glass remains an enigma. In the Edict of Diocletian, this colourless glass is listed as “*Alexandrian*” referring to Egypt, but its origin has been ambiguous. Previous studies have found overlapping strontium and neodymium isotope ratios for Levantine and Egyptian glass. Here, we confirm these findings and show for the first time, based on glasses from the ancient city of Gerasa, that hafnium (Hf) isotopes are different in Egyptian and Levantine natron glasses, and that Sb Roman glass is Egyptian. Our work illustrates the value of Hf isotopes in provenancing archaeological glass. We attribute the striking difference in Hf isotopes of Egyptian versus Levantine glasses to sorting of zircons in Nile sediments during longshore drift and aeolian transport along the south-eastern Mediterranean coast leaving behind a less juvenile fraction.

## Introduction

The Roman glass industry underwent a massive expansion over the first century CE. At its peak it supplied not only tablewares for households across the Empire but also furnished major public buildings with many tonnes of glass for windows and mosaics^1,2^. The raw glass was made by fusing Egyptian evaporitic soda (natron) and sand to produce large glass slabs in tank furnaces with capacities of 8–20 tonnes^3,4^. These were broken up and distributed to glass workshops where the glass was remelted and shaped into objects for use^5,6^. This division of production continued until at least the ninth century, when a change from a mineral soda flux over to plant ash occurred bringing about the end of the Roman glassmaking tradition^7,8^.

**Figure 1:**
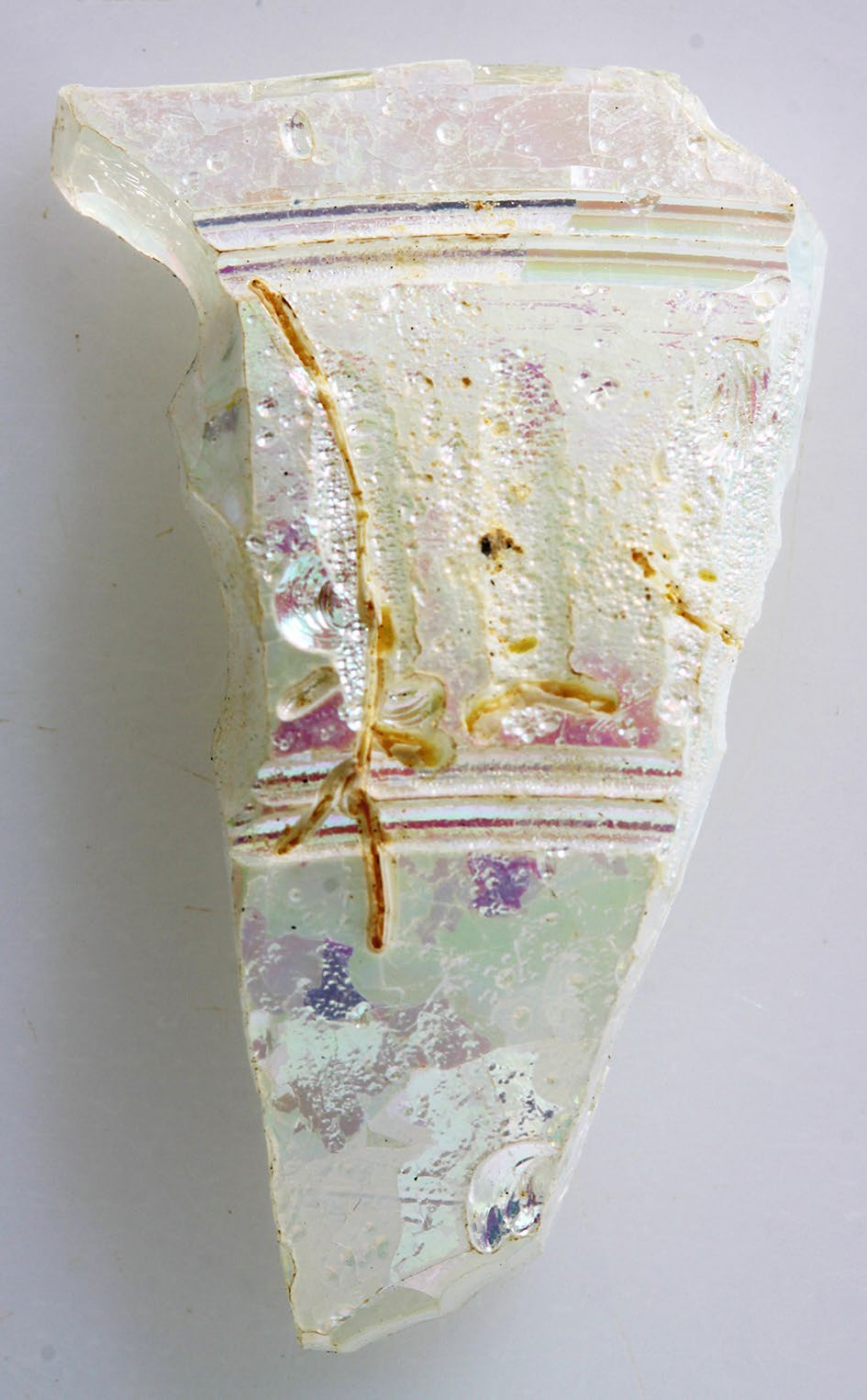
One of the colourless Roman glass sherds (J13-Ga-12-18) analysed in this study. Purple splashes are iridescence due to weathering. Photo: Danish-German Jerash Northwest Quarter Project.

The technological achievements of the Roman glass industry were precocious and not surpassed until the rise of the European industries in the eighteenth century. In particular, the Romans produced large quantities of an expensive and highly valued glass, described by Pliny^9^ as “*colourless or transparent, as closely as possible resembling rock crystal*” (Fig. 1), where the iron from the sand was oxidised from blue Fe^2+^ to very pale Fe^3+^ by the addition of antimony oxide, Sb_2_O_3_^10,11^. In the Price Edict of Diocletian, this colourless glass is listed as “*Alexandrian*” thereby referring to Egypt^12^. Despite this, the production site for this so-called Sb Roman glass is unknown but several authors have suggested, on the basis of circumstantial evidence, that it was in Egypt^13,14^ (see Supplementary Information for details).

Strong evidence that the primary glassmaking factories melting sand and natron to glass were predominantly located along the coast of the eastern Mediterranean is provided by isotopic measurements. Strontium (Sr) isotope compositions for the majority of natron glass groups are close to that of modern seawater, indicating the incorporation of marine shell in the batch and suggesting the use of beach sand as a silica source^15–17^. With regards to neodymium (Nd) isotopes, nearly all natron glass types show a characteristic Nile-related signature reflecting the use of coastal sands along the south-eastern Mediterranean that comprise largely Nile-derived sediments transported here by longshore drift^18,19^. Hafnium (Hf) isotopes have not previously been applied to man-made archaeological material (see Supplementary Information). Here, we present Sr, Nd and Hf results on natron glass types and show that, unlike the Sr and Nd systems, hafnium isotopes distinguish between natron glass made in Egypt and those made in the Levant, and, in particular, place the production of Sb Roman glass in Egypt.

**Figure 2:**
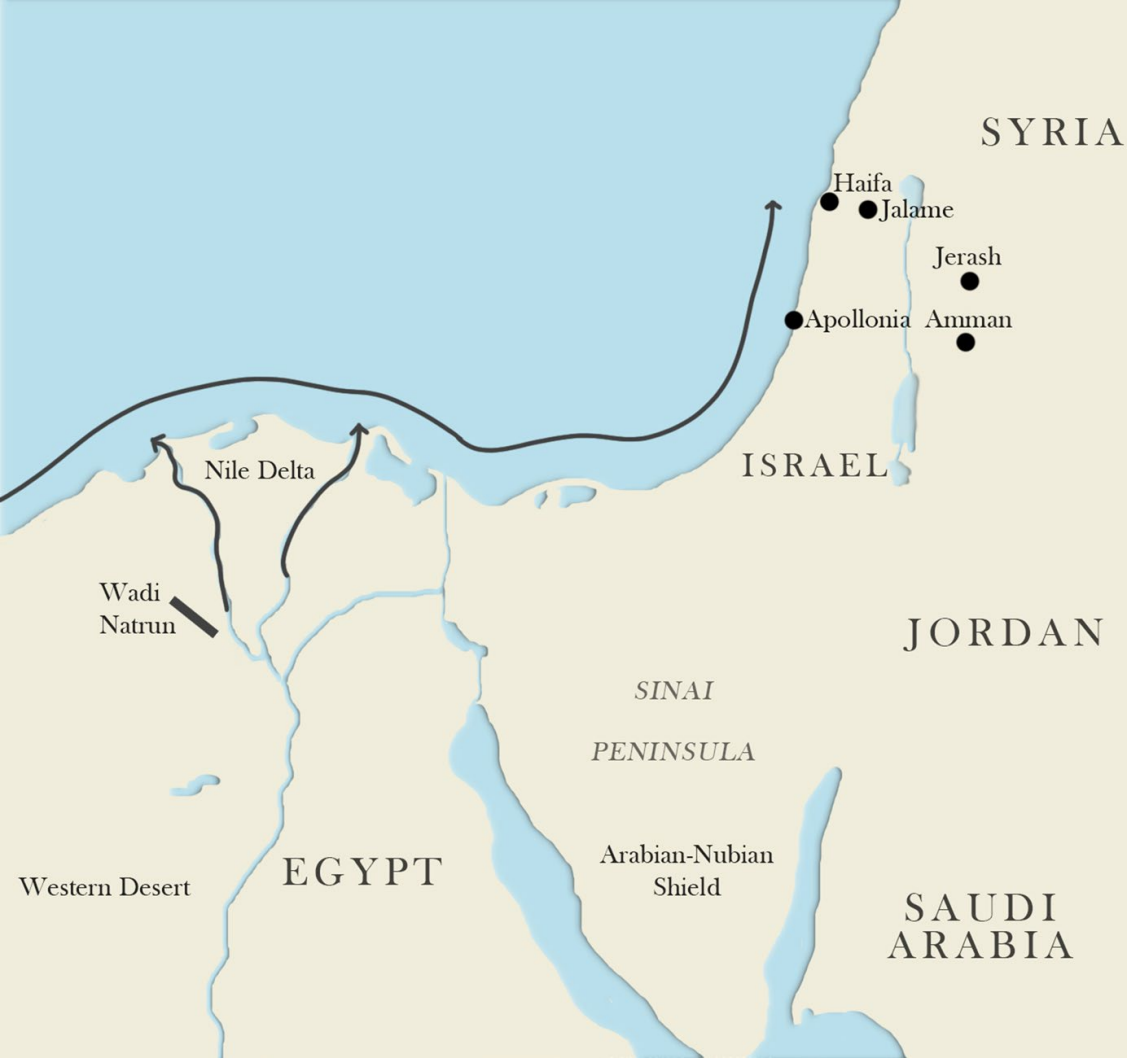
Map showing the locations of Gerasa (Jerash), N. Jordan, glass production sites at Apollonia and Jalame in the Levant and Wadi Natrun close to Nile Delta. The Blue Nile and Atbara (south of map) bring minerals to the delta from volcanics to the south in Ethiopia, which controls the Nd isotopic compositions of Nile sands. Hafnium isotopic compositions of Nile sands are instead controlled by zircons presumably dominated by erosion products of the Arabian–Nubian shield. From the delta, the Nile sands are transported by long-shore and aeolian drift along the south-eastern Mediterranean coast (black arrows). Map created by Lianna Hecht using Lightroom Classic CC/Lightroom 7.0 and Adobe Photoshop CC 2019 (×20).

The modern town of Jerash, located about 50 km from modern Jordan's capital Amman (ancient Philadelphia), is the location of the ancient city of Gerasa, which belonged to the Decapolis, a group of semi-autonomous Greco-Roman city states operating under Roman protection^20^ (Fig. 2). The city prospered during the first millennium CE until an earthquake in 749 CE led to its demise and abandonment^21,22^. Samples for this study come from excavations undertaken by the Danish-German Jerash Northwest Quarter Project in highest area within the ancient walled city where our previous elemental and Sr isotope analyses of 25 glass vessel sherds showed a dominance of Apollonia-type glass from the Syro-Palestinian Coast dating to the Byzantine period along with a small early Roman glass assemblage^23,24^.

Our screening of a further 160 glass fragments shows the presence of a larger number of previously-established compositional groups: Mn Roman and Levantine-I glass types from Syro-Palestine as well as high TiO_2_ Egypt-Ib, Egypt-Ic and Foy 2.1 types from Egypt. Two additional identified types, Sb Roman and Sb-Mn Roman glass, cannot be unambiguously attributed to either Syro-Palestine or Egypt. The latter glass type, Sb-Mn Roman glass, shows characteristics of both Roman glass types because it is the result of mixing Sb Roman and Mn Roman type glasses during recycling^25^. On the basis of our screening, a subset of 37 sherds from Gerasa that includes representatives of all identified natron glass types was chosen for Sr, Nd and Hf isotopic analysis.

## Methods

Dissolution and ion exchange chromatography were performed for 20 mg fresh glass collected from the centre of the vessels to avoid exposed surface contamination. Strontium, neodymium and hafnium isotope analyses were done by Multicollector-ICPMS at AGiR platform using a DSN nebulizer. Hafnium fractions were run in 2% HNO_3_–1% HF, mass fractionation corrected for by normalising to ^179^Hf/^177^Hf of 0.7325 and the results normalised to our in-house Ames Hf standard that was adjusted to the low Hf intensity of the glass solutions (down to 20 ppb total Hf). Neodymium and strontium analyses were corrected by normalisation to ^146^Nd/^144^Nd = 0.7219 and ^86^Sr/^88^Sr = 0.1194 and to the JNdi and NBS 987 standards, respectively. Well-characterized glass and basalt standards were processed and run with the samples to characterise reproducibility and accuracy. For major and trace elements, 1mmx1mm fresh glass fragments were mounted in epoxy, polished and analysed by electron microprobe and Laser Ablation ICPMS. See Supplementary Information for detailed description of our methods and SI Table S2 for analytical data.

## Results and discussion

**Figure 3:**
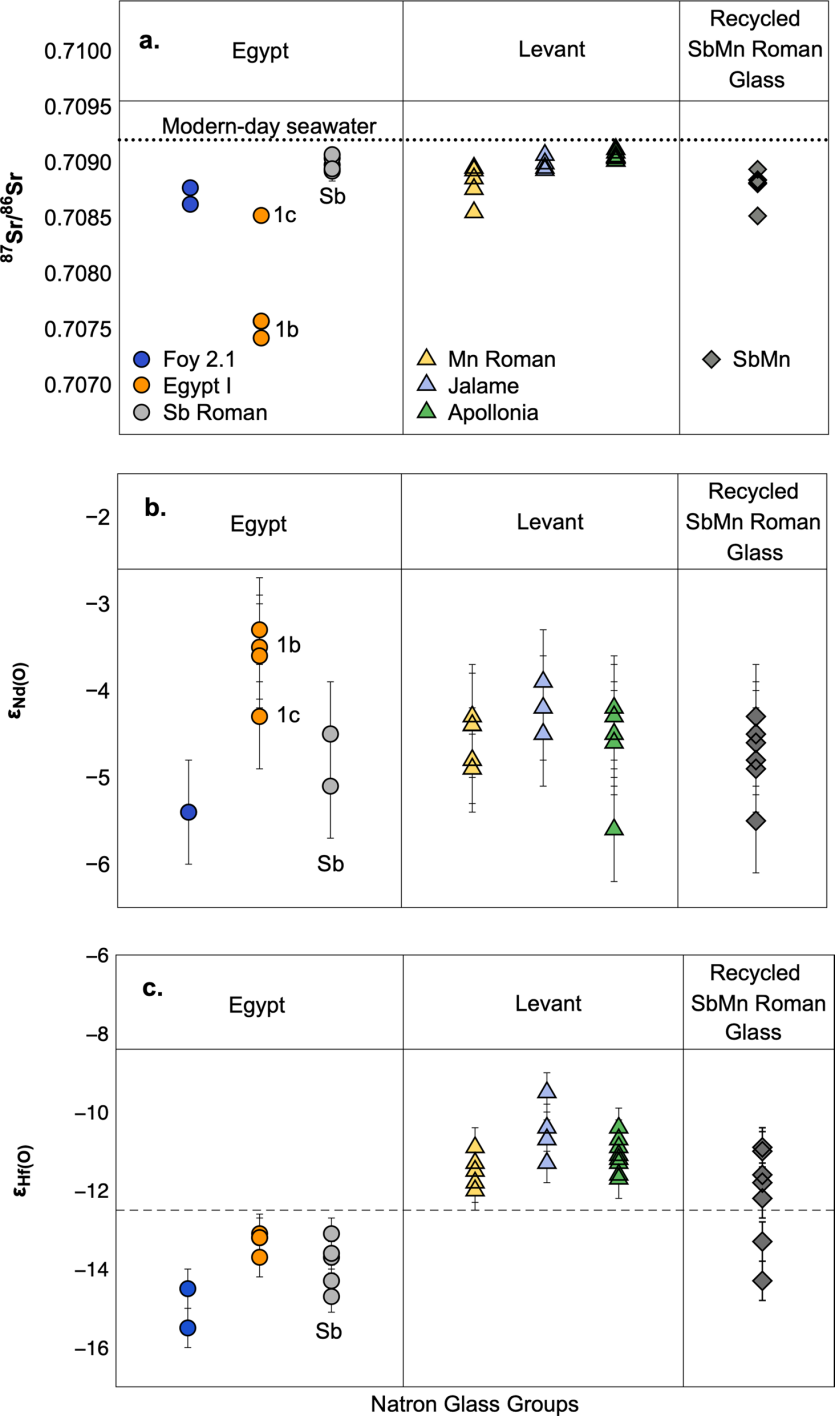
Plots illustrating (**a**) ^87^Sr/^86^Sr ratios, (**b**) ε_Nd(0)_ and (**c**) ε_Hf(0)_ values for glass types from the NW Quarter, Gerasa, N. Jordan. ε_Hf(0)_ and ε_Nd(0)_ are calculated using present-day CHUR values of 0.282785 and 0.51263, respectively^44^. Two sigma analytical precision (2σ) for ^87^Sr/^86^Sr is 0.000018 (SI Table S2), estimated from repeated run of SRM 987 Sr standard (n = 44) and is significantly smaller than symbols. 2σ for ε_Nd_ and ε_Hf_ are ± 0.4 and ± 0.5 ε units, respectively, estimated from repeat analysis of the JNdi Nd standard (n = 37) and AU Ames Hf standard (n = 25), except in cases where internal precision for individual samples was higher (SI Table S2). Samples are divided into types from Egypt (Panel 1: Foy 2.1, Egypt Ib and Ic; circle symbols), Levant (Panel 2: Mn Roman, Jalame, Apollonia; tringle symbols) as well as recycled mixtures of Sb Roman and Mn Roman glasses (Panel 3: Sb-Mn Roman glass; diamond symbols). Sb Roman glass is included with the Egyptian types based on the similarity in ε_Hf(0)_. (**a**) ^87^Sr/^86^Sr ratios for glass types plot close to modern-day seawater (0.7092; black dotted line) except for Egypt Ib-type with markedly lower ratios. (**b**) ε_Nd(0)_ values are between − 6 and − 3 for all groups and largely overlap within uncertainty. (**c**) ε_Hf(0)_ values for Egyptian and Levant glasses are distinct with the former below and the latter above − 12.2 (grey dotted line). Sb Roman glasses (grey circles in panel 1) have ε_Hf(0)_ around − 14 indistinguishable from Egypt I and Foy 2.1 glasses. SbMn Roman glasses (panel 3) have ε_Hf(0)_ values ranging from − 10 to − 14 consistent with their interpretation as mixtures of Egyptian and Levant glass types.

Sr, Nd and Hf isotope compositions of the Gerasa glasses are presented in Fig. 3 as Egyptian groups (panel 1), Levant groups (panel 2) and recycled Roman glass (panel 3). We include Sb Roman glass with the Egyptian glass groups on the basis of our new Hf isotope data (see discussion below). Nd and Hf isotope compositions are reported using the conventional ε_Nd(0)_ and ε_Hf(0)_ notations that show part per 10,000 deviations from the present-day chondritic uniform reservoir (CHUR) values^26^ (see Fig. 3 caption and Supplementary Information for details). The ^87^Sr/^86^Sr ratios for all glass types fall within a narrow range (0.7085–0.7091) close to modern-day seawater^27^ (Fig. 3a). The only exceptions are Egypt Ib glasses with markedly lower ^87^Sr/^86^Sr ratios (≈ 0.7075). Likewise, ε_Nd(0)_ values for all glass types overlap within analytical uncertainty (Fig. 3b), while ε_Hf(0)_ for Egyptian and Levant glasses are clearly distinct with the former below and the latter above − 12.2 (grey dotted line in Fig. 3c). The ε_Hf(0)_ values around − 13.9 for Sb Roman glasses place this type with Egyptian products and are indistinguishable from Egypt I and Foy 2.1 glasses. A critical observation from Fig. 3c is that the ε_Hf(0)_ values observed for Sb-Mn Roman glass encompass the entire Egypt and Levant range (panel 3 in Fig. 3c) as would be expected for mixtures of glass from Egypt (Sb Roman) and the Levant (Mn Roman). Hf isotopes in natron glass of unknown provenance therefore fingerprint whether the glassmaking sands were from Egypt or the Levant, and place Sb Roman glass production in Egypt.

To illustrate the underlying processes responsible for the difference in the Hf isotope signatures of Egyptian and Levant glasses, we begin by considering how trace elements, ^87^Sr/^86^Sr and ε_Nd(0)_ compositions of our Egyptian and the Levant type glasses from Gerasa cannot be utilise to unambiguously distinguish sand sources on the coasts of Egypt and Syro-Palestine.

The locations of the raw glass furnaces so far discovered occur mainly on the coastal strip of Syro-Palestine (e.g. Apollonia and Jalame in Fig. 2). Published evidence for primary glass furnaces in Egypt is limited, apart from those close to the ancient soda sources around the Wadi el Natrun, some 50 km northwest of Cairo^4^ (Fig. 2). Because of this paucity of known Egyptian production sites, and restrictions on the availability of Egyptian cultural material for analysis, attribution of glass types to Egypt is generally inferred from (1) a failure to match the elemental compositions of the well-characterised products of the Palestinian furnaces and (2) the elevated TiO_2_ concentrations, which are characteristic of the limited data on Egyptian sands as well as of well-provenanced Egyptian glass dating to the Islamic period^28,29^. However, this approach does not exclude potential sand sources in other areas of the Mediterranean where Nd isotopic compositions and Ti concentrations are broadly consistent with the inferred Egyptian glass compositions^19,30^. It would also fail for any glass made in Egypt using high quality sands, which had been intentionally selected to be low in iron oxides (and thus unlikely to have elevated TiO_2_) such as the sands used in the renowned antimony-decolourised glass.

The ^87^Sr/^86^Sr ratios just below the value for Holocene seawater observed for the Gerasa glasses conform to previous observations for natron glass and reflect the presence of present-day marine carbonates in the glassmaking recipe^16–18^ (Fig. 3a, SI Fig. S1). Slightly low radiogenic ^87^Sr/^86^Sr ratios of 0.7085 for one Mn Roman and one Sb-Mn sample are likely due to minor contamination by strontium from the Mn-ore added to decolourise the glass^16,31–33^ (Fig. 3a). Even lower ^87^Sr/^86^Sr ratios around 0.7075 for the Egypt Ib samples can be explained by relative high contributions of strontium from minor minerals in the glassmaking sands due to a low carbonate component in the glasses (as reflected by their low CaO concentrations; SI Table S1). Irrespective of these minor variations, the homogeneous ^87^Sr/^86^Sr ratios in glass types from the two regions exclude strontium isotopes as a discriminant between glass from Egypt and the Levant.

Hafnium and neodymium in natron glass are controlled by minerals in the sands used for glass production. A complication in distinguishing sands along the south-eastern Mediterranean coast is their common origin from the Nile Delta. The Nile drains large and widely different terranes producing sediments that accumulate in the Delta and from here are transported due to the Nile littoral cell by longshore drift around the south-eastern Mediterranean and, to a smaller degree, via aeolian transport to the coasts of Sinai and modern-day Israel^34,35^ (Fig. 2). The two major Nile tributaries, the Blue Nile and Atbara, carry mafic minerals (in particular pyroxene) high in neodymium from Tertiary basalts in the Ethiopian highlands^36^ (Fig. 2). The result is the slightly negative ε_Nd(0)_ values observed for Nile delta and coastal sands as well as in Egyptian and Levantine glass^14,16,18,19^ (Fig. 3b; SI Fig. S1). Slightly higher concentrations of Nd in Egyptian natron glass (8–11 ppm) versus Levantine glass (5–8 ppm) indicate the partial loss of these mafic minerals during longshore transport^37^, while the ε_Nd(0)_ values remain constant (SI Table S2).

Hafnium in Nile sands and thus natron glass originates from the mineral zircon that traces the detrital quartz component^38^. The Nile, Sinai and Red Sea follow a collision zone (the northern end of the East African orogeny) that marked the closure of east and west Gondwana and consisted of oceanic island arc volcanics with back-arc sedimentary basins, in some periods mixed with older crustal materials^36^. Extensive work has shown that zircons and quartz in Nile sands derive from detrital rocks that formed from the breakdown of these collision-zone terranes. The source rocks have been suggested to be the Cambrian-Ordovician sandstone covering much of North Africa^35^ or the Um Had Conglomerate although the latter is mainly made up of material eroded only from the Arabian-Nubian Shield^39^ (Fig. 2). As observed for the minerals controlling neodymium, zircon drops out of the sediments during longshore transport^34^, which is reflected in the Hf concentrations of 2–4 ppm for the Egyptian natron glass versus below 2 ppm in the Levantine glass from Gerasa (SI Table S2). An important implication of our study is therefore that the longshore transport of the Nile sediments not only leads to lower Hf concentrations in the sediments (and thus glass) along the Levantine coast, but also to changes in the Hf isotope composition. This could be due to (1) the addition of zircons of different compositions delivered by rivers which drain inland Israel or (2) a preferential deposition of larger, non-juvenile zircons during longshore transport. The first possibility can be excluded since the inland lithologies from modern Israel are dominated by carbonates, while siliciclastic sediments of Jordan drain eastwards rather than towards the Mediterranean coast^40^. Therefore, it appears that there is a progressive change in the Hf isotopic composition of eastern Mediterranean coastal sand due to hydraulic sorting of zircons of different ages and size. Unfortunately, Hf isotope data for bulk sands to confirm this have not been reported from the Nile Delta and Sinai-Israeli coasts. Fieldings et al.^41^ report values of − 15 to − 22 (average of − 18) for 5 bulk aeolian sands from the Western Desert (WD-C samples in their Fig. 1), which match well the ε_Hf(0)_ of − 16 to − 13 observed for Egyptian glass groups but their ε_Nd(0)_ values and location suggest that they are unlikely to have supplied abundant material to the sands of the eastern Mediterranean coast.

While Hf isotope studies of bulk sands are limited, numerous studies have utilised combined U–Pb dating and Hf isotopes of the detrital zircon populations in Nile sands from the Egyptian and Israeli coasts to constrain the sediment source(s). These show identical ε_Hf(0)_ overall systematics with a dominance of 0.56–1.15 Ga zircons with ε_Hf(0)_ of + 12 to − 70 representing a mixture of juvenile and non-juvenile late Mesoproterozoic to Neoproterozoic sources, as well as small populations of Archaean–Palaeoproterozoic and Palaeozoic zircons^35,39,41^. However, these studies target cores and only sometimes include analysis of rims from single, often zoned zircon grains^39,41–43^ and cannot be directly related to bulk sand compositions. Thus, analysis of bulk Nilotic sands would be required to evaluate the fractionation mechanism proposed here. For the present, we conclude that natron glass groups reflect the sorting of zircons during the longshore transport of glassmaking sands leading to a change in Hf isotope compositions along the Mediterranean coast. This feature of the coastal sands has enabled us to confirm suspicions that the famous colourless glass of ancient Rome was indeed produced in Egypt despite its low TiO_2_, Zr and Hf concentrations. The reason for the latter characteristics is most likely that iron-poor sands were targeted for their production and that these sands had zircons that were not yet sorted due to longshore transport (and thus were located in Egypt). Hafnium isotopes are likely to become increasingly important in tracing the products of the early glass industries, not only in Roman empire, but also elsewhere.

## Data availability

All data are supplied in this article, Supplementary Information and Supplementary Data Tables.

## Supplementary information

Supplementary file1

Supplementary file2

Supplementary file3
