# Supplementary material for: ‘Alexandrian’ glass confirmed by hafnium isotopes
Source: Sci Rep. 2020 Jul 9;10:11322. doi: 10.1038/s41598-020-68089-w (PMC7347594; doi:10.1038/s41598-020-68089-w)
Supplement: Supplementary file 3 — Supplementary file3 [file 41598_2020_68089_MOESM3_ESM.pdf]

SUPPLEMENTARY INFORMATION FOR

'ALEXANDRIAN' GLASS CONFIRMED BY HAFNIUM ISOTOPES

Gry H Barfod[1,2*] Ian Freestone[3], Charles E Lesher[1,2], Achim Lichtenberger[4], Rubina Raja[2,5]

[1]Aarhus Geochemistry and Isotope Research (AGiR) Platform, Department of Geoscience, Aarhus University, DK-8000 Aarhus C, Denmark

[2]Centre of Excellence Urban Network Evolutions, Aarhus University, DK-8260 Højbjerg, Denmark

[2]Department of Geoscience, Aarhus University, DK-8000 Aarhus C, Denmark

[3]Institute of Archaeology, London's Global University UCL, London, United Kingdom

[4]Institut für Klassische Archäologie und Christliche Archäologie, Münster University, Münster, Germany

[5]School of Culture and Society, Aarhus University, DK-8000 Aarhus C, Denmark

*corresponding author; grybarfod@geo.au.dk

Previous work on Natron glass provenance from Sr and Nd isotopes

It has been known for some time that Nd and Sr isotopes observed for the natron glass supplying the Roman and Byzantine empires place their production along the coast of the eastern Mediterranean[1,2]. Elemental characteristics have furthermore shown that this production must have taken place at few coastal sites in the Levant and Egypt, where the sands had ideal calcium carbonate content in the form of shells and where natron flux from the Wadi Natrun valley could easily arrive by sea. Along the Levantine coast, tank furnaces from glass 'factories' have revealed a Roman production of manganese (Mn) decolourized glass at Jalame and Haifa during the 4th century followed by Levantine glass production during Byzantine times at Apollonia during the 6th-7th centuries and at Bet Eli'ezer in the latter part of the 8th-9th century[3-5]. Low titanium (Ti), zirconium (Zr) and, most importantly for our study, Hf contents in these glasses make them distinct from natron glass types known to have been produced in Egypt; Egypt I & II, Foy 2.1 & 3.2 and HIMT glass types. Unlike the situation for the Levant, the exact locations of the production sites of these Egyptian glass groups remain largely unknown and their Egyptian origins have largely been inferred from their relatively high Ti and Zr (as well as Hf) concentrations; characteristics inherited from the Egyptian sands used in their production[6]. In contrast to this, the production location of the widely spread Roman Sb glass type has remained an enigma. The Romans produced large quantities of this highly desired, colourless glass, where the iron from the sand was oxidized from blue Fe^{2+} to very pale Fe^{3+} by the addition of antimony (Sb) oxide[7]. At a first glance, the chemistry of Sb Roman glass (in particular low Ti, Zr and Hf concentrations) differs only slightly from known Levantine products. However, despite this, it has been proposed by several studies to have been a product of an Egyptian factory for 3 main reasons; 1. the glass type have features that relate it to the sand source used for Egyptian Foy 3.2 type glasses[8,9] (see summary in Freestone et al.[10]), 2. colourless glass mentioned by Pliny as the most expensive and desirable type of glass[11,12] is referred to in the Price Edict of Diocletian[13] as Alexandrian and 3. some of the glass from furnaces in the Wadi Natrun, Egypt, is chemically very similar to the Roman antimony glass composition[14,15] (see summary in Paynter and Jackson[12]). However, this evidence is largely circumstantial and a robust chemical discriminant between Levantine and Egyptian glasses is needed.

Hafnium (Hf) isotopes have to our knowledge never been applied to pyrotechnically produced archaeological materials and only previously been used for sourcing sandstone grinding tools[16]. Here, we show that hafnium isotopes offer a new tool to provenance natron glass and in particular resolve the origin of Roman colourless glass. We begin by considering the previously published isotope data for natron glass and the current status of the understanding of their provenance. This work focused initially on elemental systematics and later expanded to strontium (Sr), neodymium (Nd) and, most recently, boron (B) isotopes. The latter traces the natron flux and is homogeneous in all glass made with natron from Wadi Natrun[17]; it is therefore beyond the scope of this paper and will not be discussed further.

Figure S1 compares Sr and Nd isotope data for natron glass groups compiled from the literature. The data were screened to eliminate the effects of glass recycling and thus potential mixing of Levant and Egypt-type signals. We therefore excluded Sb-Mn Roman glass (classified by their significant concentrations of both Sb and Mn), which is considered to be a mixture and only include end-member Mn and Sb-decolourised glasses. For Byzantine glass, only raw glass from the glass factories at Apollonia and Bet Eli'ezer in the Levant are included. Before drawing conclusions from Figure S1a, it is important to critically evaluate how well each data point can be said to be known. Given that Sr isotope analysis from TIMS and MC-ICPMS analysis has 'typical' two standard deviation (2δ of ± 20 ppm (illustrated by the red shaded band around the black line), it is reasonable to conclude that mean Sr isotopic ratios for any two samples must differ by more than 20 ppm to be truly different. Applying this principle, the most important observations from Figure S1a are (1) the overlap (≈ 0.7085 - 0.7093) of natron glass groups from the Levant (Mn Roman, Apollonia & Bet Eli'ezer glass) with Roman Sb glass, and (2) $^{87}Sr/^{86}Sr$ values for all groups just below that of modern-day seawater Sr ($^{87}Sr/^{86}Sr \approx 0.7092$; vertical black line). These observations correspond well to the general consensus that strontium isotopes in most natron glass closely resemble that of modern seawater due to the natural occurrence of shells from marine organisms in the glassmaking beach sands or addition of marine shells to the sands to obtain the ideal glass composition[18]. Given the overlap between the groups, Sr isotopes cannot be used to exclude a Levantine or Egyptian origin for Sb Roman glass.

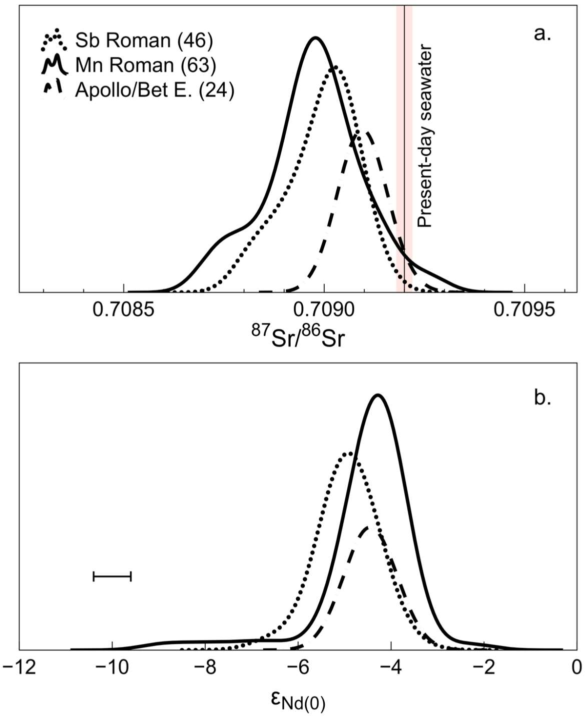

Figure S1. Kernel density distribution for a. $^{87}Sr/^{86}Sr$ ratios (bandwidth = 0.00005) and b. $\varepsilon_{Nd(0)}$ (bandwidth = 0.5) for Mn Roman glass and raw glass from glass factories at Apollonia and Bet Eli'ezer on the Levant coast as well as for Sb Roman glass suggested to have been produced in Egypt. Data compiled from Degryse[2], Ganio[38], Ganio et al.[39,40], Degryse et al.[41-43], and Freestone et al.[44]. To remove influence from potential mixing of Levant and Egypt glass due to recycling, data was screened to only include Mn Roman samples with Sb < 0.05 wt% and Sb Roman samples with Mn < 0.05 wt%. Vertical black line in S1a shows average modern-day seawater value of 0.7092[45] and the vertical red band represents 'typical' two standard deviation ($\pm 2\sigma = 20$ ppm) for TIMS and MC-ICPMS Sr analyses. Horizontal bar in lower-left corner of S1b represent typical two standard deviation ($\pm 2\sigma = 0.4$ ε_{Nd} units) for Nd analyses. For consistency, all Nd isotope data were re-calculated using present-day CHUR value of 0.51263[29].

In the same way, figure S1b shows a compilation of published Nd isotope data for Mn Roman, Apollonia, Bet Eli'ezer and Sb Roman glass. The horizontal black bar illustrates 'typical' analytical two standard deviation ($2\delta = \pm 0.4$ ε_{Nd} units) showing that $\varepsilon_{Nd(0)}$ of the two Roman glass types and the raw glass from the factories in the Levant range between -6 - -3 and, like $^{87}Sr/^{86}Sr$, overlap within uncertainty (Fig. S1b). The $\varepsilon_{Nd(0)}$ values for all groups are consistent with control from sands derived ultimately from the Nile. However, the transport of Nile sands into the Mediterranean leads to the identical Nd isotopic signals in the coastal sands from Egypt and the Levant. Nd isotopes therefore cannot be used to exclude a Levant or Egyptian origin for Sb Roman glass. While Sb and Nd systematics provide invaluable indications that glass was made on the south-eastern Mediterranean coast, their limitation is the poor discrimination between glass made in primary furnaces on the Levantine coast and that made in Egypt. It is in this context that we investigate the potential of Hf isotopes.

Hafnium in the Nile sands would be almost entirely controlled by zircon; a mineral absent in the East African volcanics that control neodymium. Instead, zircons ultimately derived from inland basement rocks are likely to dominate the hafnium signature on the Egyptian coast.

Analytical methods and results

Our samples come from the ancient city of Gerasa (today the modern city of Jerash) in northern Jordan, that prospered during the first millennium CE and belonged to the Decapolis, a group of semi-autonomous Greco-Roman cities operated under Roman rule, until an earthquake in 749 CE led to the demise and abandonment of the city[19-21]. A large selection of glass sherds (160) were analysed by Electron Microprobe (EMP) for major and minor elements and Laser-Ablation (LA)-ICP-MS for trace elements. From the 160 glass analyses, we identified a subset of 34 glass sherds for Sr, Nd and Hf isotopic analyses with the objectives of 1. including all observed natron glass types and 2. testing the disturbance on the isotope systematics from ancient recycling processes.

Table S1. Mean major element compositions (wt%) by EMP and Zr concentrations (ppm) by LA-ICP-MS for natron glass groups from the Northwest Quarter, Gerasa.

	Levant 1-2th cent		Levant 3-4th cent		Levant 5-6th cent		Recycled 1-3rd cent		Egypt? 1-2th cent		Egypt 4th cent		Egypt 2-4th cent		Egypt 2-4th cent
	**Mn Roman (4)**	*std dev*	**Jalame (4)**	*std dev*	**Apollonia (8)**	*std dev*	**SbMn Roman (7)**	*std dev*	**Sb Roman (2)**	*std dev*	**Foy 2.1 (2)**	*std dev*	**Egypt IB (3)**	*std dev*	**Egypt IC (1)**
**SiO_2**	67.63	*2.50*	67.76	*1.24*	72.71	*1.14*	67.07	*0.76*	69.63	*1.37*	65.51	*1.00*	71.28	*0.07*	70.28
**TiO_2**	0.06	*0.01*	0.07	*0.01*	0.08	*0.01*	0.09	*0.01*	0.08	*0.01*	0.17	*0.01*	0.54	*0.03*	0.28
**Na_2O**	17.64	*1.47*	15.98	*0.95*	13.74	*0.68*	16.44	*0.39*	18.98	*0.11*	18.58	*1.49*	16.62	*0.20*	15.86
**K_2O**	0.66	*0.30*	0.80	*0.37*	0.57	*0.22*	0.91	*0.20*	0.36	*0.03*	0.63	*0.25*	0.49	*0.02*	0.94
**CaO**	8.12	*1.24*	9.67	*1.55*	7.73	*0.96*	8.97	*0.91*	5.92	*1.28*	7.27	*1.37*	2.94	*0.15*	6.54
**Al_2O_3**	2.39	*0.10*	2.96	*0.05*	3.12	*0.10*	2.54	*0.06*	1.78	*0.10*	2.55	*0.37*	4.27	*0.12*	3.68
**Fe_2O_3**	0.38	*0.05*	0.46	*0.11*	0.43	*0.04*	0.62	*0.08*	0.38	*0.05*	1.14	*0.13*	1.83	*0.03*	1.30
**MgO**	0.63	*0.09*	0.70	*0.06*	0.59	*0.06*	0.73	*0.06*	0.52	*0.09*	1.26	*0.25*	1.01	*0.02*	0.89
**MnO**	0.77	*0.71*	0.09	*0.09*	0.02	*0.00*	0.98	*0.44*	0.02	*0.01*	1.42	*0.44*	0.04	*0.01*	0.08
**Sb_2O_5**	BDL	-	BDL	-	BDL	-	0.21	*0.06*	0.67	*0.06*	0.06	*0.03*	BDL	-	BDL
**CuO**	BDL	-	BDL	-	BDL	-	0.02	*0.00*	BDL	-	BDL	-	BDL	-	0.03
**P_2O_5**	0.13	*0.03*	0.13	*0.07*	0.10	*0.10*	0.19	*0.06*	0.05	*0.01*	0.13	*0.06*	0.09	*0.01*	0.22
**Cl**	0.91	*0.12*	0.87	*0.30*	0.90	*0.04*	0.81	*0.15*	1.28	*0.05*	0.99	*0.25*	0.94	*0.02*	0.78
**PbO**	0.05	*0.00*	0.06	*0.01*	0.05	*0.01*	0.06	*0.01*	0.06	*0.01*	0.05	*0.00*	0.04	*0.00*	0.05
**Pb**															
**Zr - ppm**	38	4	42	4	46	3	49	5	46	1	95	9	192	9	116

Table S1 lists the average elemental compositions of the glass types determined for the 34 sherds analysed by electron microprobe (EMP) and laser ablation (LA)-ICP-MS. Small glass chips (1mm x1mm) of fresh glass from each sample were mounted in epoxy and polished. Care was taken to avoid glass surfaces given the well-known Na loss at surfaces due to the exposure[22]. Major and minor element analyses were done on a JEOL JXA8500F with five wavelength dispersive X-ray spectrometers at WSA GeoAnalytical Lab using NIST glasses and natural and synthetic minerals for calibration. Analytical settings during quantitative WDS analysis were acceleration voltage of 20 kV, beam current of 10 nA, beam size 10 μm and 10

μm raster lengths. Counting peak times varied from 160 s for MgO down to 10 s for Na_2O, SiO_2 and Al_2O_3. Intensity correction was done by ZAF procedure, interference corrections following Donovan et al.[23] and background corrections following Donovan and Tingle[24]. Analytical precision was estimated by repeated analysis of Corning glass standards, CorB (n=149) and CorD (n=145) yielding better than 5% for element concentrations above 0.2 wt% and better than 20% for PbO below 0.5 wt%, NaO_2 below 1.5 wt% and TiO_2 below 0.1 wt%[25]. Each sample analysis is an average of six repeats. Trace element analysis was done at AGiR (Aarhus Geochemistry and Isotope Research) platform using an Agilent 7900 quadrupole ICP-MS coupled to a Resonetics 193 nm laser ablation instrument. Laser settings were laser energy at 80 mJ, 300 um line scans, 60 um spot size, 10 Hz repetition rate and acquisition time of 71 sec. Data reduction was done with offline using USGS glass standard GSE-G1 as calibration standard and by matching Si counts for samples to the SiO_2 concentrations obtained from EMP analyses. Repeated analysis of USGS glass standard GSD-G1 (n=18) was within 5% of known values for most elements.

For Sr, Nd and Hf isotope analysis of the 34 sherds (Table S2), about 20 mg glass without alteration surfaces was weighed into Teflon beakers. Dissolution involved repeated steps in 5:1 conc. HF:HNO_3 mixture followed by 6 N HCl steps. Separations of Sr, Nd and Hf were done in 5 column steps. First column step removed Fe from the dissolved sample on AG1-X8 resin. Hafnium was purified on Eichrom® Ln-spec followed by elution with HCl-HF dilutions. The fractions containing Sr and LREE from Ln-spec column were split on AG50-X8 columns. Rubidium and barium were removed from Sr on Eichrom® Sr-spec using conventional elution schemes with nitric acid dilutions[26]. Last column step was isolation of Nd from Sm using dilute HCl solutions on Ln-spec resin. Isotope measurements were done on the Nu Plasma II MC-ICP-MS using a DSN nebulizer at AGiR. During Sr isotope analysis, mass fractionation on $^{87}Sr/^{86}Sr$ ratios was corrected to $^{86}Sr/^{88}Sr$ = 0.1194 and interferences of ^{87}Rb on ^{87}Sr and ^{86}Kr on ^{86}Sr monitored by measuring signal on masses 85 (=^{85}Rb) and 84 (=^{84}Kr+^{84}Sr). These signals were less than a few mV. Repeated static measurements of the NBS 987 standard over the duration of the study yielded an average $^{87}Sr/^{86}Sr$ ratio of 0.710362 (2δ, n=22). This standard was run after every four samples and the $^{87}Sr/^{86}Sr$ ratios of the samples normalized to its accepted value of 0.710248[30]. Normalized $^{87}Sr/^{86}Sr$ ratios of 0.703479 ± 0.000015 (2δ) were obtained for standards USGS standards BHVO-2, which is within uncertainty of $^{87}Sr/^{86}Sr$ value

for BHVO-2 of 0.703481 ± 16 reported by Weis et al.[31]. The mean $^{87}Sr/^{86}Sr$ ratio for Corning B was 0.708989 ± 0.000013 (n=10). For Nd isotopic analysis, glass samples and standards were corrected for mass fractionation by normalizing to a natural $^{146}Nd/^{144}Nd$ ratio = 0.7219. Isotopes of Sm, Ba and Ce were monitored for potential interferences.

Table S2. Sr, Nd & Hf isotope compositions of natron glass from Northwest Quarter, Geresa.

Sample Number	Sr - ppm	$^{87}Sr/^{86}Sr$	Nd - ppm	$^{143}Nd/^{144}Nd$	$\varepsilon_{Nd(0)}$	Hf - ppm	$^{176}Hf/^{177}Hf$	$\varepsilon_{Hf(0)}$
**Mn Roman**								
J13-Ha/Hal-13-47	572	0.708925 (18)	7.27	0.512378 (21)	-4.9	0.89	0.282460 (15)	-11.5
J14-Lc-71-1	581	0.708548 (18)	6.67	0.512393 (21)	-4.6	1.04	0.282445 (15)	-12.0
J15-Jj-2-9	468	0.708757 (18)	5.69	0.512394 (21)	-4.6	0.89	0.282452 (15)	-11.8
J13-Ha/Ha1-14-19 S1	536	0.708853 (18)	6.79	0.512419 (21)	-4.1	1.00	0.282477 (25) ⋆	-10.9
**Jamale type**								
J14-Kb-3-12	-	0.708987 (18)	-	0.512440 (21)	-3.7	-	0.282516 (15)	-9.5
J15-Ob-13-15 B	536	0.709064 (18)	6.91	0.512424 (21)	-4.0	1.00	0.282490 (17)	-10.4
J15-Qc-18-3	538	0.708948 (18)	7.36	0.512413 (21)	-4.2	1.14	0.282466 (15)	-11.3
J14-Ih-24-5 S2	453	0.708928 (18)	6.51	0.512405 (21)	-4.4	1.06	0.282482 (15)	-10.7
**Apollonia type**								
J13-Fi-0-14	415	0.709013 (18)	6.79	0.512405 (21)	-4.4	1.20	0.282455 (15)	-11.7
J14-Ld-50-12	464	0.709085 (18)	7.05	0.512423 (28)*	-4.0	1.13	0.282483 (15)	-10.7
J14-Li-70-3 A	454	0.709051 (18)	6.88	0.512421 (21)	-4.1	1.21	0.282469 (15)	-11.2
J15-Pe-5-48 A	549	0.709116 (18)	7.93	0.512419 (21)	-4.1	1.24	0.282490 (15)	-10.4
J14-Ke-3N-401	464	0.709048 (18)	7.56	0.512404 (21)	-4.4	1.16	0.282464 (25) ⋆	-11.3
J14-Li-77-5	416	0.709034 (18)	6.62	0.512417 (21)*	-4.2	1.11	0.282471 (15)	-11.1
J14-Ke-28-4	409	0.709042 (18)	6.54	0.512352 (21)	-5.4	1.08	0.282478 (16)	-10.9
J14-Li-50-101 B	411	0.709010 (18)	6.67	0.512418 (21)	-4.1	1.43	0.282457 (17) ⋆	-11.6
**SbMn Roman**								
J13-Ha/Ha 1-13-50 BS	688	0.708516 (18)	7.72	0.512391 (21)	-4.7	1.40	0.282382 (15)	-14.3
J15-Nb-57-133 A	564	0.708935 (18)	6.91	0.512416 (21)	-4.2	1.16	0.282475 (15)	-11.0
J15-Jl-32-3 S1 A	503	0.708837 (18)	6.54	0.512408 (21)	-4.3	1.07	0.282477 (15)	-10.9
J15-Jl-32-3 B	512	0.708815 (18)	6.86	0.512403 (21)	-4.4	1.19	0.282439 (15)	-12.2
J14-Jc-67-4	520	0.708805 (18)	6.97	0.512390 (21)	-4.7	1.21	0.282456 (15)	-11.6
J14-Jc-62-11	477	0.708841 (18)	6.41	0.512354 (21)	-5.4	1.21	0.282408 (15)	-13.3
J14-Jd-32-219	508	0.708811 (18)	7.01	0.512387 (21)	-4.7	1.24	0.282452 (15)	-11.8
**Sb Roman**								
J14-Jd-43-4 S1	511	0.708919 (18)	6.18	0.512405 (21)	-4.4	1.20	0.282396 (15)	-13.7
J14-Lbd-21-6	375	0.708976 (18)	5.30	0.512377 (21)	-4.9	1.13	0.282402 (15)	-13.6
J13-Ga-12-18#	331	0.70894 (11)*	-	-	-	-	0.282415 (12)	-13.1
J13-Ga-12-13##	-	0.709035 (18)	-	-	-	-	0.282381 (12)	-14.3
J15-Rbcd-9-1##	-	0.709067 (18)	-	-	-	-	0.282371 (12)	-14.7
**Foy 2.1**								
J14-Kg-3D-411	535	0.708769 (18)	8.51	0.512359 (21)	-5.3	2.21	0.282375 (15)	-14.5
J15-Of-27-1 S1	826	0.708621 (18)	8.03	0.512359 (21)	-5.3	2.43	0.282346 (15)	-15.5
**Egypt Ib**								
J14-Kc-3-59	211	0.707418 (21)	9.93	0.512466 (21)	-3.2	4.54	0.282415 (15)	-13.1
J14-Kg-3-375	224	0.707567 (18)	10.4	0.512460 (21)	-3.3	4.73	0.282412 (15) ⋆	-13.2
J14-Kg-40-3	228	0.707570 (18)	10.5	0.512455 (32)*	-3.4	4.64	0.282399 (15) ⋆	-13.7
**Egypt Ic**								
J14-Kh-51-3 S1	337	0.708520 (18)	9.24	0.512416 (21)	-4.2	2.88	0.282411 (15)	-13.2

⋆ repeated analysis (n=3)
Major and trace elements for this sample is reported in Barfod et al., 2018
Sb Roman glass from NW Quarter determined visually and major element compositions by micro-XRF
* Analytical error based on combining Sr analyses from 2015 and 2020.

The JNdi standard yielded a $^{143}Nd/^{144}Nd$ ratio of 0.512060 ± 0.000022 (2δ) corresponding to 42 ppm and all samples were bracketed by this standard and normalized to its known values of $^{143}Nd/^{144}Nd$ = 0.512108. Repeated analysis of AU Nd Ames std (n=14) also run with the

samples yielded $^{143}Nd/^{144}Nd$ of 0.511964±18 (2δ), which is close to our longterm reproducibility of 0.511968 ±18 (2δ). Likewise, USGS basalt standards BIR-1 (n=3) yielded 0.513083 ± 0.000017 (2δ) close to the Georem recommended value of 0.513091 ± 0.000014. Glass standard Corning B (n=13) from Corning Museum of Glass yielded $^{146}Nd/^{144}Nd$ of 0.512138 ± 0.000055 (2δ). This standard has to our knowledge not previously been characterized for Nd isotopic composition and due to its very low Nd concentrations, it was only run once at 20 ppb. Hafnium fractions were dissolved in 2% HNO_3-1% HF and mass fractionation corrected for by normalizing to the known natural ratio of $^{179}Hf/^{177}Hf$ of 0.7325[27]. The intensity of our in-house Ames Hf standard was adjusted and run at three solution levels to bracket the intensities of the low Hf intensities of the glass solutions. During the course of the runs, the in-house AU Hf Ames yielded 0.282184 ± 0.000012 (2δ) corresponding to 41 ppm. All results are reported relative to the known value of $^{176}Hf/^{177}Hf$ of 0.282167 for this standard, which is isotopically very close to the JMC-475 Hf standard (0.28216) developed by Patchett & Tatsumoto[27]. Using this correction, repeated analysis of JMC-475 (n=15) throughout the run yielded a mean $^{176}Hf/^{177}Hf$ ratio of 0.282165 ± 0.000015 (2δ). USGS basalt standards BHVO-2 (n=9) yielded 0.283104 ± 0.000010 (2δ) identical to the Georem recommended value of 0.283104 ± 0.000010 and BCR-2 (n=3) 0.282878 ± 0.000013 (2δ) corresponding well to the Georem recommended value of 0.282865 ± 0.000013. Glass standard Corning B (n=13) from Corning Museum of Glass[28], which has not previously been characterized for $^{176}Hf/^{177}Hf$ yielded 0.282212 ± 0.000015 (2δ). Throughout the session ^{172}Yb, ^{175}Lu, ^{181}Ta and ^{182}W signals were monitored for potential interferences. These signals were all less than a few mV. Epsilon Hf and Nd were calculated from present-day CHUR values of 0.512630 and 0.282785[29] (Table S2).

In accordance with our previous work on glass recovered from the Northwest Quarter in Geresa[34], we observe a dominance of 'Levantine-I' type products from the Palestine-Syria Coast. We use the term Levantine-I to refer to Byzantine glasses with Al_2O_3/SiO_2 between 0.038-0.05 and TiO_2/Al_2O_3 ratios below 0.05[9] and further divide this group broadly into 'Apollonia' and 'Jalame' types on the basis of their Na_2O/SiO_2 and CaO/Al_2O_3 ratios (Phelps et al.[33] in their fig. 7b) - and the often elevated MnO concentrations in the Jalame types[3] (Table S1). Besides this Byzantine glass, we found Mn Roman, Sb-Mn Roman and Sb Roman glass

types, but only identified glass types from Egypt (Foy 2.1[34] and Egypt-Ib & Ic[35]) and Sassanian plant ash glass among finds from the remains of a private house destroyed during the 749 CE earthquake[36]. This is unlikely to be a coincidence; their preservation here suggests that the trade connections to Egypt and the east were in place and active, but that, once broken, the glass ended up in the recycling pile and the chemical signatures were diluted and lost due to the large quantities of Levantine material.

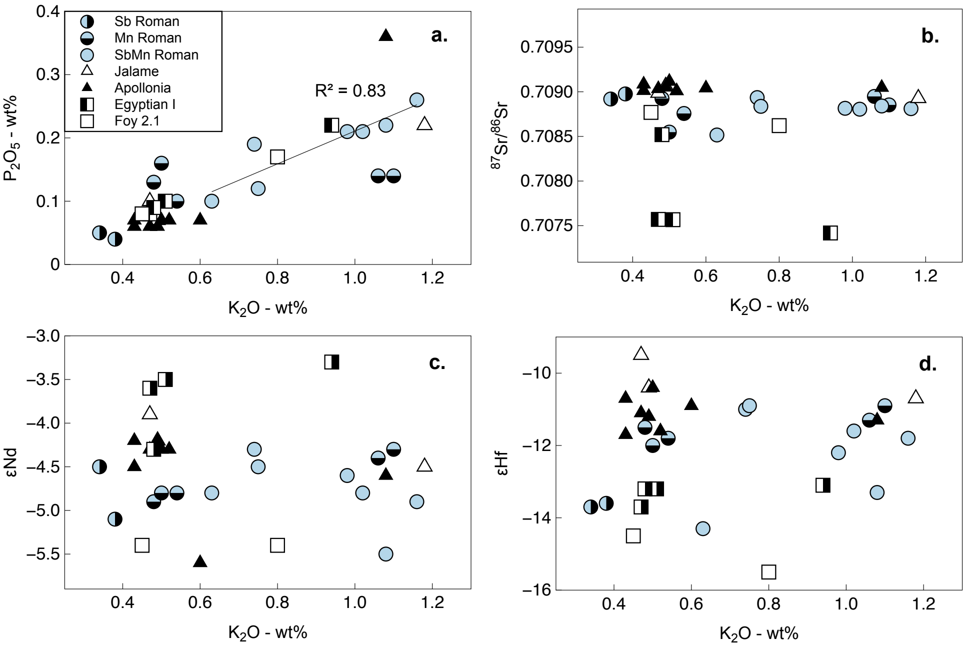

Fig. S2. Index of recycling (K_2O) versus a. P_2O_5, b. $^{87}Sr^{86}Sr$, c. $\varepsilon_{Nd}(0)$ and d. $\varepsilon_{Hf}(0)$ for natron glass from NW Quarter, Jerash.

Test of the potential influence on isotope systematics from repeated remelting was done by selecting a number of Sb-Mn Roman glass and 'Levantine I' type glass characterized by low and high K_2O and P_2O_5 contents corresponding to a low and high degrees of recycling, respectively. The reason is that fuel ash and vapour contaminate with K_2O and P_2O_5 in particular and samples with high concentrations of these elements would be therefore expected to have been repeatedly recycled (Fig. S2). Strong correlations between K_2O and P_2O_5 reflecting this process have been observed at several sites[32,37]. Following this, we observe a strong correlation between K_2O and P_2O_5 (Fig. S2a), but no correlation between isotope ratios and these indexes of recycling (Fig. S2b-d). We thus regard the effect of remelting on Sr, Nd and Hf isotope compositions of the Levant and Egyptian natron glass groups negligible, and consider the signal from Hf isotopes as well as Sr and Nd isotopes as robust.

References

1. Freestone, I. C., Leslie, K. A., Thirlwall, M. & Gorin-Rosen, Y. Strontium isotopes in the investigation of early glass production: Byzantine and early Islamic glass from the Near East. *Archaeometry*, **45**, 19-32 (2003)

2. Degryse, P. (ed) *Glass Making in the Greco-Roman World: Results of the ARCHGLASS project* **4**. (Leuven University Press, 2014).

3. Brill, R. H. Scientific investigations of the Jalame glass and related finds. In *Excavations at Jalame: site of a glass factory in late Roman Palestine*, 257-294 (1988).

4. Freestone, I. C., Jackson-Tal, R. E. & Tal, O. Raw glass and the production of glass vessels at late Byzantine Apollonia-Arsuf, Israel. *J. glass stud.*, 67-80 (2008).

5. Freestone, I. C. The provenance of ancient glass through compositional analysis. *MRS Online Proceedings Library Archive* **852** (2004).

6. Foy, D., Picon, M., Vichy, M. & Thirion-Merle, V. Caractérisation des verres de la fin de l'Antiquité en Méditerranée occidentale: l'émergence de nouveaux courants commerciaux. *Échanges et commerce du verre dans le monde antique*, 41-85 (2003).

7. Sayre, E.V. The intentional use of antimony and manganese in ancient glasses. In *Advances in glass technology. Part 2: history papers and discussions of the technical papers of the VI International Congress on glass*, 263-282 (1963).

8. Maltoni, S., Silvestri, A., Marcante, A. & Molin, G. The Transition from Roman to Late Antique Glass: New Insights from the Domus of Tito Macro in Aquileia (Italy). *J. Archaeol. Sci.* **73**, 1–16 (2016).

9. Schibille, N., Sterrett-Krause, A. & Freestone, I. C. Glass Groups, Glass Supply and Recycling in Late Roman Carthage. *Archaeol. Anthrop. Sci.* **9**, 1223–41 (2017).

10. Freestone, I. C., Degryse, P., Lankton, J., Gratuze, B. & Schneider, J. HIMT, glass composition and commodity branding in the primary glass industry. *Things that travelled: Mediterranean glass in the first millennium ce* (ed. D. Rosenow) 159-190 (2018).

11. Barag D. Recent important epigraphic discoveries related to the history of glassmaking in the Roman period. In: *Annales de l'Association Internationale pour l'Histoire du Verre* **10** (Madrid and Segovia, 1985) 109–116 (1987).

12. Paynter, S. & Jackson, C. Clarity and brilliance: antimony in colourless natron glass explored using Roman glass found in Britain. *Archaeol. Anthrop. Sci.* **11**, 1533-1551 (2019).

13. Whitehouse, D. Glass in the Price Edict of Diocletian. *Glass Studies,* **46**, 189-191 (2004).

14. Picon, M., Thirion-Merle, V. & Vichy, M. Les verres au natron et les verres aux cendres du Wadi Natrun (Egypte). *Bulletin de l'Association Française pour l'Archéologie du Verre* **22**, 36-41 (2008).

15. Thirion-Merle V. & Vichy M. Note sur la composition chimique des verres de l'épave des Embiez. In: *Revuearchéologique de Narbonnaise* **40**, 266–268 (2007).

16. Burton, M. M., Muniz, A. A., Abbott, P. L., Kimbrough, D. L., Haproff, P. J., Gehrels, G. E. & Pecha, M. Sourcing sandstone cobble grinding tools in southern California using petrography, U–Pb geochronology, and Hf isotope geochemistry. *J. Archaeol. Sci.* **50**, 273-287 (2014).

17. Devulder, V., Vanhaecke, F., Shortland, A., Mattingly, D., Jackson, C. & Degryse, P. Boron isotopic composition as a provenance indicator for the flux raw material in Roman natron glass. *J. Archaeol. Sci.* **46**, 107-113 (2014).

18. Brems, D., Ganio, M., Latruwe, K., Balcaen, L., Carremans, M., Gimeno, D., Silvestri, A., Vanhaecke, F., Muchez, P. & Degryse, P. Isotopes on the beach, part 1: strontium isotope ratios as a provenance indicator for lime raw materials used in Roman glass-making. *Archaeometry* **55**, 214-234 (2013).

19. Lichtenberger, A., & Raja, R. New archaeological research in the Northwest quarter of Jerash and its implications for the urban development of Roman Gerasa. *Am. J. Archaeol.* **119**, 483-500 (2015).

20. Raja, R. & Lichtenberger, A. The archaeology and history of Jerash. 110 years of excavations. An introduction. In *Archaeology and History of Jerash. 110 Years of Excavations,* 1-6 (Brepols Publishers 2018).

21. Raja, R., & Lichtenberger, A. Defining Borders: The Umayyad-Abbasid Transition and the Earthquake of AD in Jerash. *Byzantine and Umayyad Jerash*. *Transitions, Transformations, Continuities*, 265-286 (2019).

22. Jackson, C.M., Greenfield, D. and Howie, L.A., 2012. An assessment of compositional and morphological changes in model archaeological glasses in an acid burial matrix. *Archaeometry* **54**, 489-507.

23. Donovan, J. J., Snyder, D. A. & Rivers, M. L. An improved interference correction for trace element analysis. *Microb. Anal.* **2**, 23-28 (1993).

24. Donovan, J. J. & Tingle, T. N. An Improved Mean Atomic Number Correction for Quantitative Microanalysis. *J. Microsc-Oxford* **2**, 1-7 (1996).

25. Adlington, L. W. The Corning Archaeological Reference Glasses: New Values for" Old" Compositions. *Papers from the Institute of Archaeology* **27** (2017).

26. Pin, C. & Bassin, C. Evaluation of a strontium-specific extraction chromatographic method for isotopic analysis in geological materials. *Anal. Chim. Acta* **269**, 249-255 (1992).

27. Patchett, P. J. & Tatsumoto, M. A routine high-precision method for Lu-Hf isotope geochemistry and chronology. *Contrib. Mineral. Petr.* **75**, 263–267 (1981).

28. Brill, R. H. *Chemical analyses of early glasses*. Corning, NY: Corning Museum of Glass (1999).

29. Bouvier, A., Vervoort, J. D. & Patchett, P. J. The Lu–Hf and Sm–Nd isotopic composition of CHUR: constraints from unequilibrated chondrites and implications for the bulk composition of terrestrial planets. *Earth Planet. Sci. Lett.* **273**, 48-57 (2008).

30. Ohr, M., Halliday, A. N. & Peacor, D. R. Sr and Nd isotopic evidence for punctuated clay diagenesis, Texas Gulf Coast. *Earth Planet. Sci. Lett.* **105**, 110-126 (1991).

31. Weis, D., Kieffer, B., Maerschalk, C., Barling, J., de Jong, J., Williams, G. A., Hanano, D., Pretorius, W., Mattielli, N., Scoates, J. S. & Goolaerts, A. High-precision isotopic characterization of USGS reference materials by TIMS and MC-ICP-MS. *Geochem. Geophy. Geosy.* **7** https://doi.org/10.1029/2006GC001283 (2006).

32. Barfod, G. H., Freestone, I. C., Lichtenberger, A., Raja, R., & Schwarzer, H. (2018). Geochemistry of Byzantine and Early Islamic glass from Jerash, Jordan: Typology, recycling, and provenance. *Geoarchaeology* **33**, 623-640 (2018).

33. Phelps, M., Freestone, I. C., Gorin-Rosen, Y. & Gratuze, B. Natron glass production and supply in the late antique and early medieval Near East: The effect of the Byzantine-Islamic transition. *J. Archaeol. Sci.* **75**, 57-71 (2016).

34. Foy, D., Picon, M., Vichy, M. & Thirion-Merle, V. Caractérisation des verres de la fin de l'Antiquité en Méditerranée occidentale: l'émergence de nouveaux courants commerciaux. *Échanges et commerce du verre dans le monde antique*, 41-85 (2003).

35. Schibille, N., Gratuze, B., Ollivier, E. & Blondeau, É., 2019. Chronology of early Islamic glass compositions from Egypt. *J. Archaeol. Sci.* **104**, 10-18 (2019).

36. Lichtenberger, A., Raja, R., Eger, C., Kalaitzoglou, G. & Sørensen, A. H. A newly excavated private house in Jerash. Reconsidering aspects of continuity and change in material culture from Late Antiquity to the early Islamic period. *Antiquité Tardive* **24**, 317-359 (2017).

37. Tal, O., Jackson-Tal, R. E. & Freestone, I. C. Glass from a Late Byzantine secondary workshop at Ramla (south), Israel. *J. Glass Stud.* **50**, 81–95 (2008).

38. Ganio, M. A 'true' Roman glass: evidence for primary production in Italy. PhD Thesis, KU Leuven (2013).

39. Ganio, M., Boyen, S., Fenn, T., Scott, R., Vanhoutte, S., Gimeno, D., Degryse, P., 2012a. Roman glass across the Empire: an elemental and isotopic characterization. Journal of Analytical Atomic Spectrometry 27, 743-753.

40. Ganio, M., Boyen, S., Brems, D., Scott, R., Foy, D., Latruwe, K., Molin, G., Silvestri, A., Vanhaecke, F., Degryse, P., 2012b. Trade routes across the Mediterranean: a Sr/Nd isotopic investigation on Roman colourless glass. Glass Technology: European Journal of Glass Science and Technology A 53, 217-224

41. Degryse, P., Schneider, J., Lauwers, V. & Brems, D. Sr-Nd isotopic analysis of glass from Sagalassos (SW Turkey). *J. Cultural Heritage* 9, e47-e49 (2008).

42. Degryse, P., Henderson, J. & Hodgins, G. Isotopes in vitreous materials, a state-of-the-art and perspectives. *Isotopes in Vitreous Materials*, 15-30 (2009).

43. Degryse, P., Schneider, J., Lauwers, V., Henderson, J., Van Daele, B., Martens, M., Huisman, H.D.J., De Muynck, D. & Muchez, Ph. Neodymium and strontium isotopes in the provenance determination of primary natron glass production. *Isotopes in Vitreous Materials*, 53-72 (2009).

44. Freestone, I. C., Leslie, K. A., Thirlwall, M. & Gorin-Rosen, Y. Strontium isotopes in the investigation of early glass production: Byzantine and early Islamic glass from the Near East. *Archaeometry* **45**, 19-32 (2003).

45. Veizer, J., Ala, D., Azmy, K., Bruchschen, P., Buhl, D., Bruhn, F., Carden, G. A. Diener, A., Ebneth, S., Godderis, Y. & Jasper, T. $^{87}Sr/^{86}Sr$, $\delta^{13}C$ and $\delta^{18}O$ evolution of Phanerozoic seawater. *Chem. Geol.* **161**, 59-88 (1999).
